# Assessing Low-Mineralized Surface Water via *Danio rerio* Embryo Toxicity Test: A Case Study of Glacial Lakes in the Northern Tien Shan

**DOI:** 10.3390/biology15191718

**Published:** 2026-09-30

**Authors:** Mariya A. Suvorova, Anna S. Nizkorodova, Alina A. Dmitriyeva

**Affiliations:** 1 Department of Genetics and Molecular Biology, Faculty of Biology and Biotechnology, Al-Faraby Kazakh National University, Almaty 050040, Kazakhstan; dmitriyeva.alin@gmail.com; 2 M. Aitkhozhin Institute of Molecular Biology and Biochemistry, Almaty 050012, Kazakhstan; anna_niz@yahoo.com

**Keywords:** high-altitude lakes, zebrafish embryos, developmental effects, gene expression, Northern Tien Shan

## Abstract

Bioassays are rarely used in ecotoxicological studies of high-altitude lakes located above 3000 m a.s.l., as ultra-low mineralization of water poses a major obstacle to toxicity assessment in biological objects. For the first time, we conducted a 96 h bioassay of surface water from supraglacial, proglacial and moraine-dammed lakes of two distinct mountain ranges of the Northern Tien Shan (average elevations of 3000–3600 m a.s.l.) using *Danio rerio* embryos. The study demonstrates the feasibility of using the *D. rerio* embryo toxicity test to survey the initially low-mineralized surface water of high-altitude lakes. For several Ile Alatau lakes, analysis of lethal and sublethal endpoints (including molecular biomarkers *cyp1a*, *xpc*, and *dpolb* gene expression) demonstrated a mild toxic effect, whereas physicochemical analysis failed to reveal contamination exceeding maximum permissible concentrations. In the Dzungarian Alatau, significant pollution with total petroleum hydrocarbons was detected in the lake cascade beneath the remote Tuyte Glacier. An altitude-dependent effect on zebrafish embryos was observed, ranging from moderate to no toxicity. The obtained results indicate that *D. rerio* embryo toxicity test might be applied to ecotoxicological studies of ultra-fresh water sources supplied by the melting mountain cryosphere, thus simplifying, reducing the cost of, and expanding the monitoring of remote high-mountain areas.

## Simple Summary

The study addresses the challenge of assessing water pollution in remote high-mountain lakes, where ultra-low mineral content typically hinders biological assays. The primary objective was to demonstrate the feasibility of using zebrafish (*Danio rerio*) embryos—a common biological test object—to evaluate the water quality of glacial lakes. Nine lakes sourced by melting glaciers and snowpack were studied across two mountain ranges, Ile Alatau and Dzungarian Alatau, of the Northern Tien Shan. The mountains are situated in Central Asia, at the intersection of the borders of Kazakhstan, China and Kyrgyzstan borders. For the first time, significant water pollution with petroleum products was detected in very remote glacial lakes of Dzungarian Alatau. By evaluating survival and malformation rates, we showed that zebrafish embryos are able to maintain normal development when incubated in extremely low-mineralized but natural water of glacial lakes. The bioassay allows the revealing of the toxicity of water pollutants below permissible levels established by Sanitary Standards. The analysis of target gene expression proposes the presence of some uncommon agent(s) that is not detected by general physicochemical analysis. These findings demonstrate that zebrafish embryo assay offers a reliable and cost-effective approach for monitoring high-mountain water sources, ultimately helping to protect downstream water supplies and manage fragile alpine ecosystems.

## 1. Introduction

Bioassay of natural water sources is the essential part of ecotoxicological monitoring applied to indicate the aquatic pollution and quantify the degree of its impact on test organisms. In water toxicity assessment, bioassay approaches based on acute test on algae, macrophytes, invertebrates, and fish are commonly used, combined into a battery of bioassays. A set of lethal and sublethal endpoints is applied, improved and continuously reassessed for the accurate evaluation of the water quality. The acute toxicity test on zebrafish (*Danio rerio*) embryos is an internationally standardized method commonly used in aquatic ecotoxicology [1,2]. Basically mortality and sublethal endpoints such as hatching alterations and morphological abnormalities are evaluated; behavioral biomarkers, molecular and biochemical approaches to assess genotoxicity, oxidative stress and xenobiotics metabolism are additionally added depending on the research aims [2,3,4,5,6]. Although focusing on wastewater management and toxicity of distinct xenobiotics promotes test standardization, it restrains the application of zebrafish embryo toxicity assay to natural water of specific chemical composition which is assumed to affect the data output considerably.

Mountain headwaters primarily fed by melting cryosphere (snowpack, glaciers, permafrost) hold ultra-fresh, low-mineralized water which represents extreme conditions for zebrafish development and is considered to confound the toxicity results independently of chemical pollution. The specificity of water composition substantially restricts the bioassay research, besides the remoteness, arduity and complicated drainage basins of high-altitude ecosystems pose major obstacles to field investigations. Glaciated mountain catchments have an outstanding role as principal sources of fresh water, especially in arid regions (so called “water towers”). In Eurasia glaciers are concentrated in the Alps in Europe, in the Himalayas in Asia and in the Pamir and Tien Shan mountains in Central Asia. Whereas environmental research is attributed mostly to Alps, Tibetan Plateau and less to Himalayas, studies concerning the pollution status of Tien Shan are extremely scarce. It is now well established that mountain cryosphere is contaminated by both legacy and current-use organic and inorganic pollutants [7]. The main evidenced cryosphere contaminants are: black carbon, fallout radionuclides, heavy metals, persistent organic pollutants and recently microplastics [8,9,10,11,12,13,14,15]. The primary source of pollution in remote mountain areas is the long-range atmospheric transport of pollutants originating from anthropogenic emissions, which are precipitated and initially accumulated in the mountain snowpack [16].

At the same time, the pollution and toxicity status of high-mountain glacierized catchments as primary collectors of meltwater runoff remains considerably understudied. High-altitude lakes, as primary catchments for snowmelt and glacial runoff, are predominantly studied in relation to Glacial Lake Outburst Flood (GLOF) events and glacier retreat. However they represent promising targets for ecotoxicological research in high-mountain areas. Recently anthropogenic pollution has been detected in remote alpine lakes of the Tibetan Plateau [17,18], the Alps [19,20], and the Hindu Kush Himalayas [21]. The assessment of pollution is performed by chemical analytical methods, since they are situated in nival zone where severe climatic conditions and low mineralization determine extremely low productivity. Few bioindication studies on macroinvertebrates, diatoms, and zooplankton are concentrated on lakes located lower, in the alpine zone [22,23]. Eventually, direct assessment of high-mountain water toxicity to aquatic organisms is only achievable using bioassay approach.

The Tien Shan mountain system is located in the center of Eurasian continent, stretching predominantly east-west across northwest China, south Kazakhstan, Kyrgyzstan and Tajikistan, its cryosphere is the primary and crucial source of freshwater in those extremely arid regions [24,25]. Generally Tien Shan mountain system is subdivided into Western, Central, Northern and Eastern Tien Shan. The valleys and lowlands of the Northern Tien Shan (NTSh), which geographically spans China, Kazakhstan and Kyrgyzstan, are densely inhabited, intensively explored and are considered to be a vital transportation hub linking China and Central Asian countries. NTSh holds many mountain ranges among which the largest and most important are Kyrgyz Alatau, Ile Alatau, and Dzungarian Alatau. The Ile Alatau range is the principal climate-and-landscape forming system in South-East Kazakhstan; its northern lowlands are occupied by the large and intensively growing Almaty urban agglomeration with a population of about 4 million people. Headwaters of supplying rivers all have modern glaciers lowered to 3300–3500 m a.s.l., among which the Tuyuksu glacier system is one of the most thoroughly studied mountain glaciers in the world. Dzungarian Alatau is placed along the state border between the Republic of Kazakhstan and the People’s Republic of China. Here, the glacial-nival zone lies above 3300–3600 m a.s.l., the altitudinal boundary of the permafrost belt is estimated at 2500 m a.s.l. [26], which is lower than the average for the Northern Tien Shan. In Kazakhstan, the Dzungarian Alatau is sparsely populated and in general poorly investigated compared to other Tien Shan regions. 

To date, as far as we are aware, no studies have focused on the assessment of water toxicity of high-altitude lakes using zebrafish embryos as test objects. It is generally accepted that low initial temperature and extremely low mineralization severely hinder embryonic development. Considering that optimization of incubation temperature is unavoidable in any bioassay research, and that natural water is not equal to artificial incubation media, we propose applying acute zebrafish embryo toxicity assay to survey water quality in high-altitude lakes. For this purpose, several proglacial and moraine-dammed lakes located in nival zone of Kazakhstan Tien Shan were targeted for investigation. High-altitude lakes of the NTSh are fully accessible during the late summer season, when the maximum of glacier melt occurs, presenting an opportunity to collect water samples.

Our objective was to analyze the lethal and sublethal endpoints in zebrafish embryos incubated in initial low-mineralized water of high-altitude lakes. The survival, malformation and hatching rates, as well as target gene expression (*cyp1a*, *xpc*, and *dpolb)*, were assessed after 96 h exposure. The induction of *cyp1a* expression in late embryos and larvae is generally associated with acute response to organic compounds such as PAHs (polycyclic aromatic hydrocarbons), PCBs (polychlorinated biphenyl), dioxins, and many pesticides and pharmaceuticals [6,27]. The *xpc* and *dpolb* genes are related to different types of excision repair, as we hypothesized that the amount of pollutants in high-altitude lakes may result in the activation of only the first link of DNA damage response (DDR). The *xpc* gene encodes the XPC (Xeroderma Pigmentosum complementation group C) protein, which is a part of the GG-NER (global genome nucleotide excision repair) pathway, acting as a primary sensor for many DNA-damaging adducts and binding to the undamaged DNA strand [28]. The *dpolb* gene encodes DNA-polymerase β, which is the main repair polymerase involved in the base pair repair (BER) process [29]. The expression of both of these genes has been previously studied in response to a number of mutagenic factors in fish [30,31].

Using a spatially distinct set of supraglacial, proglacial and moraine-dammed lakes of the Northern Tien Shan (all located above 3000 m a.s.l.), we demonstrated that zebrafish embryos are able to maintain normal development for 96 h in low-mineralized natural surface waters. We expect our results to advance research on zebrafish embryo adaptability and promote bioassay-based monitoring of high-altitude water sources and the mountain cryosphere.

## 2. Materials and Methods

### 2.1. Study Area and Surface Water Samples Collection

The current study investigates nine high-altitude lakes across two distinct ranges of the Northern Tien Shan, the Ile Alatau (Trans-Ile, formerly Zailiiskiy Alatau) and the Dzungarian Alatau, located along the border intersection between South-East Kazakhstan and North-East Kyrgyzstan, and North-West China (Figure 1). The details of the geographical locations and images of the studied lakes are given in Table 1 and Supplementary Materials (Figures S1 and S2).

The Ile Alatau is the northernmost range of the NTSh, the climate is continental, maximal precipitation and snow accumulation peak in high mountains are attributed to spring—early summer, whereas minimal precipitation—to the winter season, under the influence of Siberian anticyclone [32,33]. Permafrost is widespread above 3000 m; the runoff is provided by the snowmelt in June-July and glacier melt in August [32]. The following lakes located within the highly glacierized headwater catchments were surveyed: Kishi Almaty River, three lakes: (1) L. T-1 (3500 m a.s.l.), small proglacial lake beneath Molodezhniy Glacier, has underground and surface runoff, in the studied period was shallow, touristic activity is low; (2) L. M. Mametova (3550 m a.s.l.), small proglacial lake beneath M. Mametova Glacier, has no surface runoff, is continuously monitored and siphoned due to GLOF threat, popular hiking site, touristic activity is moderate and steadily increasing; (3) L. Titov (3320 m a.s.l.), moraine-dammed lake, shallow in a studied period is fed by snowpack and permafrost melt, experiences increasing direct anthropogenic pressure due to its popularity as a recreation area; Issyk River: (4) L. Akkol (3111 m a.s.l.), large moraine-dammed lake in alpine meadows, is sourced by r. Kokbulak which originates beneath the glaciers of Tesken-Kokbulak Glacial System, has no surface runoff; 3 km lower, it erupts into the Lake Bozkol in the Issyk river valley, touristic activity is moderate; Orta Turgen River, (5) L. #1 (3560 m a.s.l.), large proglacial lake beneath Gorniy Institute Glacier, is the very source of Turgen River in Turgen valley; touristic activity is very low. All rivers fed by the mountain cryosphere pass through the Almaty urban agglomeration and gather lowland into the great Ile River of the Balkhash-Alakol Water Basin.

Dzungarian Alatau is a massive, distinct mountain system, characterized by high aridity among other regions of Northern Tien Shan. Climate is continental, with dry and hot summers and dry and cold winters, influenced throughout the year by air masses formed over the western Siberia, the Turanian Plateau as well as over the Atlantic Ocean, the average annual precipitation in high altitudes ranges from 1000 to 1600 mm [26,34]. The studied area in Dzungarian Alatau includes the lake cascade beneath the Tuyte Glacier in Zhamankol range of the Central Dzungaria (Kazakhstan). Tuyte is a small, north-oriented glacier in the middle of the range and contributes to the Tekeshbulak River, which is a tributary of the large mountain Kora River which belongs to Balkhash-Alakol Water Basin. The area is wild; signs of human presence are absent, lakes are unnamed and for the research purposes were labeled by the glacier name. The four lakes in cascade were monitored: (1) L. Tuyte-1 (3325 m a.s.l.), small supraglacial lake at the very body of the Tuyte Glacier, partially covered with ice, not marked on the satellite maps; (2) L. Tuyte-2 (3110 m a.s.l.), ice-contact proglacial lake; (3) L. Tuyte-3 (3068 m a.s.l.), was shallow at the time of sampling; (4) L. Tuyte-4 (3070 m a.s.l.), large moraine-dammed lake at the very end of alpine zone.

The surface water samples were collected in Ile Alatau in August–September 2024 and in Dzungarian Alatau in late July 2025, during peak glacial melting. The composite probes were collected from each lake; the limitation in the total sample volume is determined by the landscape, as all equipment and samples were hand-carried by the research team. Water sampling was conducted in accordance with State Standard ISO 5667-24 [35]. The water samples were collected into the sterile plastic sampling bottles (Y&K Health Care, Anseong, Republic of Korea) and delivered into the laboratory as fast as possible (in case of Dzungarian Alatau within two days due to sampling site remoteness). In the laboratory, water samples were filtered using PES 0.22 μm filter flasks (UltraCruz, Dallas, TX, USA) and stored at −20 °C until use. The primary measurements of principal water parameters were conducted in situ using a portable calibrated water quality tester (“Jinan Huiquan Electronic”, Jinan, China).

### 2.2. Physicochemical Analysis of Surface Water Samples

The hydrological analysis of natural water samples was conducted by treaty with the Laboratory of chemical and technical studies (accreditation #Kz.T.02.0782 of Republic of Kazakhstan) of the Institute of Hydrogeology and Geoecology of Akhmedsafin (Almaty, Kazakhstan). Analyses were performed in accordance with State sanitary-epidemiological standards for water sources for drinking water using reference GOST methods. Briefly, parameters were determined: (a) general mineralization and dry residue by the gravimetric method; (b) total hardness by complexometric titration; (c) HCO_3_^−^ by sequential acidimetric titration; (d) NO_3_^−^ by photometric method; (e) SO_4_^2−^ by complexometric titration; (f) Cl^−^ by argentometry; (g) total petroleum hydrocarbons (TPH) by the fluorometric method with the hexane extraction; (h) phenolic index by fluorometry; (i) lindane and DDT by GLC; (j) surfactants by fluorometry; (k) Ca^2+^ and Mg^2+^, by complexometric method; (l) Al, Cd, Mn, Cu, Mo, Ni, Pb, Se, Ag, Sr, Cr, Zn by ICP-MS on Agilent AA140 Atomic Absorption Spectrophotometer (Agilent Technologies, Santa Clara, CA, USA). Fluorimetric measurements were carried out on a liquid luminescent photometric analyzer (fluorimeter) “Fluorate-02-3M” (LLC LUMEX-marketing, St. Petersburg, Russian). Gas chromatography-quadrupole mass spectrometry (GC/MS) was performed on Agilent 5977C GC/MSD (Agilent Technologies, CA, USA).

### 2.3. D. rerio Embryotoxicity Test

Animal care and egg production. A breeding stock of healthy and unexposed wild-type zebrafish (*D. rerio*) was used for egg production. Mature lab-bred fish were kept in aquaria with a loading capacity of a minimum of 1 L per fish, at a water temperature of 23–26 ± 1 °C, at the Aquaria facility of Faculty of Biology and biotechnology of al-Faraby Kazakh National University (Almaty, Kazakhstan). Fish were fed twice per day with dry and frozen food. For spawning, in the evening a net of fish (1:2 female:male ratio) was transferred into the 20 L spawning aquaria with fresh prewarmed water (26 ± 1 °C); bottom was covered with a 2 mm plastic mesh to prevent egg predation by producers. After spawning, eggs were collected and transferred into the large sterile glass Petri dishes for microscopic analysis. Fertilized eggs at the late blastula-early gastrula stage at about 5 hpf (this stage allows to reject false-fertilized eggs) with no observable abnormalities were distinguished under the stereomicroscope MBS-100T (“Biolab”, Moscow, Russian) and used for the bioassay.

Embryos exposure. For the exposure, the water samples were prewarmed to 26 ± 1 °C. 25 newly fertilized eggs were randomly distributed directly into sterile Petri dishes (6 mm in diameter) filled with the natural water samples in a final volume of 20 mL per dish, and incubated at 26 ± 1 °C in the dark in incubator TS-1/20 (“Smolensk sktb spu”, Smolensk, Russia) for 96 h. Test was run in five replicates for each bioassay group under the static conditions. 50 µL 1% (*w*/*v*) methylene blue was added to each dish to prevent infection and facilitate the scoring of coagulated embryos. Each run was accompanied by the respective control of purified maintenance water. The control water matched the physicochemical parameters used for broodstock housing and spawning; the detailed water parameters are listed in the Supplementary Table S1. Key water parameters (TDS, pH, EC) were measured at the beginning and end of each bioassay run using a portable calibrated water quality meter (“Jinan Huiquan Electronic”).

Embryos were evaluated and scored for lethal and sublethal endpoints daily using stereomicroscope MBS-100T (“Biolab”) at 10×–50× magnification, and were staged according to Kimmel et al. [36]. Embryos were defined to be dead when meeting one of the following lethal endpoints: (a) embryo coagulation, (b) non-detachment of tail, (c) non-formation of somites, (d) no heartbeat, recorded after 48 hpf [1]. At 48, 72 and 96 hpf the hatching rate (hatched embryos per number of survived embryos) was evaluated. The teratogenic effects were recorded at 72 and 96 hpf (the spine malformations could be definitely distinguished after hatching), including the malformations of head and eyes, malformations of chorda and tail, yolk deformities and growth retardation (assessed by optical comparison with control embryos). The malformation rate at 96 hpf was evaluated and the share of general abnormalities as spinal curvatures, tail malformations, pericardial edema and growth delay was recorded. For detailed analysis and image acquisition, microscopes BE580T (“Ningbo Icoe Commodity Co.”, Ningbo, China) equipped with digital camera UNISON-45PA (“Ningbo Icoe Commodity Co.”) and Biolab-I (“Biolab”) equipped with digital camera CMEX-12f (“Euromex”, Duiven, The Netherlands) were used. For further gene expression analysis 96 h embryos were stored at −20 °C in 10% DMSO (*v*/*v*). The tests were considered to be valid if they met the following acceptance criteria: (a) parent fertility rate is ≥70%, (b) the survival rate in correspondent control group is ≥90% until the end of exposure, (c) the malformation rate in control group is ≤10% until the end of exposure [1].

The water toxicity levels were evaluated based on observed survival and malformation rates, applying the 10% no-observed-effect threshold and 50% effect threshold in the *D. rerio* embryos tests, as per OECD guideline [1]. Accordingly, water samples were categorized as possessing non-toxic (0–10%), slightly toxic (10–20%), moderately toxic (20–50%), toxic (50–70%) and highly toxic (70–100%) effect.

Ethical statement. All the experimental protocols used in the study were approved by the Local Ethical Committee (approval IRB-A699, 2024–11) of the al-Faraby Kazakh National University (Kazakhstan). 

### 2.4. Gene Expression Analysis

Total RNA isolation from *D. rerio* embryos. Total RNA from *D. rerio* embryos was isolated using the GenUP Total RNA Kit (“Biotechrabbit”, Berlin, Germany). For each extraction, three embryos were placed in a 2 mL microtube, two sterile zirconium beads were added, frozen at −80 °C for 30 min, and homogenized using a KZ-III-FP automated homogenizer (“Servicebio”, Wuhan, China). The following settings were used: 45 s at 70 Hz, 15 s rest, total 5 cycles at 4 °C. LB lysis buffer from the GenUP Total RNA Kit (“Biotechrabbit”) was then added and the rest of procedure followed the manufacturer’s instructions. The concentration and quality of the isolated RNA were assessed using a Nano-500 micro spectrophotometer (“Allsheng”, Hangzhou, China).

cDNA Synthesis in a Reverse Transcription Reaction. The reverse transcription reaction was performed in 20 µL of a mixture containing proprietary reverse transcription buffer (“NEB”, Ipswich, MA, USA), 200 u M-MuLV-RT (“NEB”), 625 µM dNTPs, 0.5 u RNasin, 5 µM Random Hexamers (“Thermo Fisher Scientific”, Waltham, MA, USA), 1.25 µM oligo(d)T (“Thermo Fisher Scientific”), and 5 µL of total RNA. Reaction conditions were as follows: 10 min at 25 °C, 60 min at 42 °C, and 10 min at 85 °C. The resulting cDNA was used for real-time PCR immediately or stored at −80 °C.

Quantitative PCR Analysis. qPCR was performed on a Q2000B Real-Time qPCR System (“LongGene”, Hangzhou, China) using the following protocol: 3 min at 95 °C for 1 cycle; 15 s at 95 °C and 60 s at 62 °C (fluorescence data collection) for 40 cycles; melting curve plotting from 95 °C to 60 °C. SYBR Green, component of CAPITAL qPCR Green Mix 4x (“Biotechrabbit”), was used as the fluorescent dye. The 16 µL reaction mixture contained 4 µL of CAPITAL qPCR Green Mix 4x (“Biotechrabbit”), 1.6 µL of cDNA, 143 nM of each of forward and reverse primers (for target genes) or 75 nM of each of forward and reverse primers (for the reference gene). Two qPCR reactions (two technical replicates) were performed with each obtained cDNA sample. Universal primers ccggacacggacaggattg (Fw) and cgctccaccaactaagaacg (Rv) were used to detect expression of the reference gene (18S rRNA) [37]. Primers ccaacctgcaagtgtccgatgag (Fw) and gctcctggacctctgggtagtgc (Rv) were used to detect expression of the *cyp1a* gene; *xpc* gene—ccgagtggtgggaggagactatgg (Fw) and gatgttggcaatggcttgtccag (Rv); *dpolb* gene—gaggaggagtctttacaccgccgta (Fw) and gcgtgaagcctttctctagtgcgtg (Rv). The reaction specificity was determined from the melting curve of the PCR product and data from NTC (non-template control) samples; melting curves for all studied genes are given in Supplementary (Figure S3) and prove the specificity of used primers. Also in Supplementary Materials we provide graphs of qPCR amplification efficiency for each of the genes studied, including the reference gene (Figure S4). Amplification efficiency for each primer pair was determined using a serial dilution across 5 points of pooled cDNA (*n* = 6). Amplification efficiency (*E*), calculated in the program Q-Gene96 v.4.0, ranged from 92% to 105% (slopes of −3.19 to −3.52), with correlation coefficients (R^2^) ≥ 0.99. Relative quantification of each gene expression level was normalized according to the housekeeping gene expression levels. Relative mRNA expression of the genes was calculated in the Q-Gene96 v.4.0 program according to [38].

### 2.5. Statistics

Each bioassay run has been accompanied by the corresponding control. For both experimental and control groups, the initial number of embryos was 25 per dish, in five replicates (total *n* = 125 per bioassay run). Since not all exposed embryos were used for further analysis, between 20 (for L. T-1 and L. Tuyte-1) and 40 (for control group) total RNA samples were used for gene expression analysis (considering that embryos were pooled in groups of three for RNA isolation). To unify the data comparison, the statistical analysis of all control groups was performed using the Kruskal–Wallis test, the absence of statistically significant differences among the control groups has been proved. Comparisons between two groups (one of which was control) based on the quantitative values were assessed via the Mann–Whitney U test for independent samples [39] and the Wilcoxon matched-pair signed-rank test for pair-matched samples in Stata 13.0 program (“StataCorp LP”, College Station, TX, USA). For all statistical analyses, 5% level of significance was accepted (α ≤ 0.05). The statistical test power (1-β) and effect size were calculated in G*Power Version 3.1.9.7 (Universität Düsseldorf, Federal Republic of Germany) according to Kang [40]. Interpretation of calculated effect size (Cohen’s d) was as follows: d value of 0.2 indicated a small effect, 0.5 indicated a medium-sized effect, and 0.8 indicated a large effect [41]. The correlation coefficient (*r*) was calculated in the Microsoft Excel statistical package according to [42].

## 3. Results

### 3.1. Physicochemical Assessment of Surface Water Samples from High-Altitude Lakes of NTSh

The composite water samples obtained from high-altitude lakes of Ile Alatau and Dzungarian Alatau were subjected to general physicochemical analysis. The detailed water parameters are represented in Supplementary (Table S1). General parameters and ion concentrations were referenced to Maximum Permissible Concentration (MPC) established by the State Hygienic Standards for Drinking Water. pH of all lakes was within 6.6–7.0. The chemical composition of Ile Alatau lakes is heterogeneous. All samples were transparent except for L. #1 where glacial flour has been observed. Across all studied lakes, water mineralization was low ranging from 43 mg/L (L. Titov) to 70 mg/L (L. Akkol). The orders of ion concentrations are: (a) by Ca^2+^ concentration: L. Akkol > L. #1 > L. M. Mametova > L. T-1 > L. Titov; (b) by Mg^2+^: L. M. Mametova > L. #1 = L. T-1 = L. Titov > L. Akkol; (c) by HCO_3_^−^: L. Akkol = L. M. Mametova >L. #1 > L. T-1 = L. Titov. Al^3+^ was detected in all samples with the exception of L. Akkol; Cu^2+^, Mo^2+^ and Ni^2+^ were evenly distributed; Mn^2+^ predominated in L. #1; Se^4+^ was detected in all lakes with the maximum concentration in L. Titov. In all samples, Cd^2+^ concentration was below detectable limits. Pb^2+^ levels followed the order L. Akkol > L. #1 > L. M. Mametova > L. Titov, with all concentrations below the MPC for drinking water. However, for lead the MPC for fishery water was reached and was increased 1.5-times in L. Akkol and L. #1. No traces of total petroleum hydrocarbons (TPHs), surfactants, DDT, lindane or 2,4-D were detected across any of the samples. 

Regarding the high-altitude lake cascade beneath the Tuyte Glacier (Dzungarian Alatau), the studied lakes included supraglacial L. Tuyte-1, proglacial L. Tuyte-2, intermediate L. Tuyte-3 and main moraine-dammed L. Tuyte-4. A general similarity in hydrochemical parameters was observed, characterized by extremely low mineralization and slightly acidic water (pH 6.0–6.8). All water samples were transparent except for L. Tuyte-2 which contained a significant amount of glacial flour. Across all studied lakes, the total mineralization (10–11 mg/L), Ca^2+^ and Mg^2+^ concentrations were extremely low; moreover, in L. Tuyte-1 Mg^2+^ was below the limit of detection. Ions of Mn, Ni, Cu and Zn were distributed nearly equally among the studied lakes; meanwhile Al^3+^ and Se^2+^ were not detected. Several compounds were characteristic of supraglacial L. Tuyte-1 only: trace amounts of Ag, Mo, Cr and Pb ions (at a level comparable to L. #1 of Ile Alatau, although remaining below the MPC for drinking water). The analysis revealed significant cumulative pollution of lakes by total petroleum hydrocarbons at levels significantly exceeding the MPC: L. Tuyte-1 (2.7 × MPC) < L. Tuyte-2 (4 × MPC) < L. Tuyte-3 (6 × MPC) < L. Tuyte-4 (8 × MPC). No traces of surfactants, DDT, lindane or 2,4-D were detected in any of the samples. 

### 3.2. Zebrafish (D. rerio) Embryo Toxicity Assay of Surface Water Samples from High-Altitude Lakes of NTSh 

Throughout the experiments, mortality and malformation rates for corresponding control groups did not exceed 10%, averaging 4.6% and 3.0%, respectively, thereby confirming the validity of bioassay runs. Across all groups, no statistically significant changes in survival rates between 24 hpf and 48 hpf were observed; therefore, data for 48 hpf were omitted to simplify the graphical representation. 

#### 3.2.1. Developmental Effects and Target Genes Expression in Zebrafish (*D. rerio*) Embryos After 96 h Exposure to Surface Water Samples from High-Altitude Lakes of the Ile Alatau (NTSh)

Surface water samples from five high-altitude lakes in the Ile Alatau range were assessed for developmental effects on *D. rerio* embryos over a 96 h exposure. The results of bioassay experiments are represented in Figure 2. In control groups, hatching occurred at 72–96 hpf due to the relatively low temperature of incubation (26 ± 1 °C). The overall survival rate to 96 hpf in embryos exposed to surface water of L. T-1 (84.6%, *p* = 0.07), L. M. Mametova (89.1%, *p* = 0.20) and L. Akkol (89.7%, *p* = 0.14) was lower than in the control group, in other groups, the survival rate was comparable to the control. Regarding hatching in L. T-1 and L. Akkol, it was accelerated by 72 hpf (67.3%, and 63.8%, respectively, *p* = 0.001) and successfully completed by 96 hpf, in L. #1 group the hatching rate was comparable to the control.

Delayed hatching at 96 hpf was observed in two groups: the hatching rate decreased to 84.6% (*p* = 0.002) in L. Titov and to 62.1% (*p* = 0.001) in L. M. Mametova. Across the studied samples, the malformation rates, in ascending order, were as follows: L. M. Mametova, 3.2% (*p* = 0.759) < L. #1, 8.5% (*p* = 0.032) < L. Titov, 9.8% (*p* = 0.044) < L. T-1, 12.5% (*p* = 0.004) < L. Akkol, 19.2% (*p* = 0.003). The types of malformations found were: spinal and tail curvatures, pericardial edemas, growth retardation and depigmentation. The types of malformations varied across exposure groups. Depigmentation was observed in the L. Titov group only, constituting about a quarter of all observed abnormalities, where all types of malformations were detected, with growth retardation being the most frequent. Growth retardation was a prevalent malformation in embryos incubated in surface water of lakes Akkol, T-1 and Lake #1. Pericardial edema occurred frequently in embryos incubated in surface water from L. T-1 and L. Titov, whereas no signs of edema were observed in embryos incubated in samples from L. Akkol. The spinal curvatures contributed significantly to total abnormalities in embryos exposed to water samples from Lake Akkol and Lake#1. Tail malformations were numerous in embryos incubated in samples from Lake T-1; however, they were less pronounced in other groups such as L. Titov, L. Akkol and L. #1. In the L. M. Mametova group abnormalities were limited to tail malformations (entirely) and minor pericardial edema. It should be noted that more than one malformation type can be observed in the same embryo. Representative images of malformed zebrafish embryos are shown in Figure 3.

Primers for assessing the expression level of *dpolb* and *cyp1a* genes were designed in such a way as to exclude the possibility of the influence of genomic DNA contamination: the size of the intron between exons 12 and 13 of the *dpolb* gene is 1060 nt, and between exons 3 and 4 of the *cyp1a* gene—1166 nt. The expression level of *D. rerio* target genes showed different values for the studied lakes of Ile Alatau. Of all the studied lakes, only the L. M. Mametova group showed no statistically significant differences from the control in the expression level of all three genes (Figure 2D). While L. T-1, which is located in the same gorge, showed a statistically significant increase in *xpc* gene expression (3.8 times compared to the control). Water samples from L. #1 and L. Akkol showed a statistically significant decrease in *dpolb* gene expression to 19% and 16%, respectively. Along with this, for both groups an increase in *xpc* gene expression by 2.5 times for L. #1 (significant and reliable size effect) and by 1.5 times for L. Akkol (significant, but not reliable size effect) was shown. The L. Titov group showed a statistically significant decrease in the expression of the *cyp1a* gene (up to 18%) and an 11-fold increase in the expression of the *dpolb* gene compared to the control.

#### 3.2.2. Developmental Effects and Target Gene Expression in Zebrafish Embryos After 96 h Exposure to Surface Water Samples from High-Altitude Lakes of Dzungarian Alatau (NTSh)

The surface water samples from the four-lake cascade originating beneath the Tuyte Glacier (Kora River valley, Dzungarian Alatau) were assessed for developmental effects on *D. rerio* embryos over a 96 h exposure; the results of bioassay are presented in Figure 4. The survival rate varied significantly among studied groups. For zebrafish embryos incubated in water samples from Lake Tuyte-4, the last lake in the cascade, the survival rate throughout the 96 h incubation was 98.4%, which is comparable to the control. The malformation rate in this group was insignificant, 7.3% (*p* = 0.11), the main malformation types observed were spinal curvatures. Across other groups, the survival rates by 96 hpf followed a descending order: L. Tuyte-3, 90.4% (*p* = 0.19) > L. Tuyte-2, 73.6% (*p* = 0.01) > L. Tuyte-1, 65.6% (*p* = 0.004). Notably, embryos incubated in L. Tuyte-2 sample exhibited mortality in two waves: by 24 hpf (survival rate: 84.8%, *p* = 0.01) and by 96 hpf (survival rate: 73.6%, *p* = 0.01). For the supraglacial L. Tuyte-1 group survival rates decreased gradually from 24 hpf (92.8%, *p* = 0.11) to 72 hpf (87.2%, *p* = 0.01) followed by a significant drop of 25% by the end of hatching. Overall, the hatching process was accelerated across all bioassay groups. For instance, in L. Tuyte-1 the percentage of embryos hatched by 72 hpf was 2.7 times higher than in the control group (26.3% vs. 7.66%, *p* = 0.006). In remaining groups on average, half of all embryos hatched by 72 hpf. By 96 hpf the hatching in all groups was completed successfully. The percentage of malformed embryos at 96 hpf was statistically different in three bioassay groups; however, in L. Tuyte-3 and L. Tuyte-2 the malformation rate was mild: 12.3% (*p* = 0.02) and 16.7% (*p* = 0.009), respectively. In these groups spinal curvatures affected the majority of malformed embryos, tail malformations and pericardial edema were found to be minor abnormalities. In L. Tuyte-3 growth retardation accounted for a quarter of all abnormalities; in L. Tuyte-2 they appeared less frequently. The incubation of embryos in water samples from L. Tuyte-1 for 96 h resulted in a significant teratogenic effect with the malformation rate in surviving embryos assessed at 42.6% (*p* = 0.009). Regarding malformations, growth retardation, spinal curvatures, and pericardial edemas were observed, edema frequently extended to the yolk sac, tail malformations were recorded in only a few embryos. Most malformed embryos were severely weakened after hatching and were unlikely to survive long-term. Representative images of abnormal zebrafish embryos are shown in Figure 3.

The expression level of *D. rerio* target genes during incubation in water samples from the Dzungarian Alatau lakes showed a similar pattern for three of studied lakes (Figure 4); a statistically reliable and significant increase in the expression level of the *cyp1a* gene was observed: 10-fold for L. Tuyte-1, 7-fold for L. Tuyte-2 and 5-fold for L. Tuyte-3. As for L. Tuyte-4 a statistically reliable and significant 11-fold decrease in *cyp1a* expression level was observed. A 2.4-fold increase in the expression of the *xpc* gene for L. Tuyte-2 and 16.6-fold decrease for L. Tuyte-4 was also noted (reliable and significant). As for the *dpolb* gene, a statistically reliable and significant decrease in the expression level was noted for two lakes: 4-fold for L. Tuyte-3 and 5-fold for L. Tuyte-4.

## 4. Discussion

The ecotoxicological status of high-altitude lakes remains severely underestimated despite their vulnerability to long-range atmospheric pollution. Such a gap is determined by the following limitations: (a) remoteness of study areas and difficulties in samples transportation; (b) peculiarities of water composition; and (c) low temperature of water, that together seem to repel scientific interests. The initial temperature of water samples is very low (0–10 °C), making it unsuitable for bioassay on fish, thus an increase in incubation temperature to the physiological level (26–28 ± 1 °C) is inevitable. It has been shown that the higher temperatures significantly alter the toxicity of water pollutants, elevating their solubility, reactivity rate and bioavailability, increasing uptake, transformation and elimination rate constants [43,44]. Thus, while maintaining the initial water composition, bioassay may indeed reveal the toxicity otherwise mitigated by low temperature. During incubation of *D. rerio* embryos water pH and hardness should be optimized to standard maintenance levels (pH–6.6–7.0, ºdH–100–300 mg/L CaCO_3_ [1,45], as it is generally accepted that incubation in media deficient in Ca, Mg and Cu results in lowered embryo and larvae survival rates and poor hatchability [46]. The exposure of *D. rerio* embryos to natural ultra-fresh waters with divergent ionic composition is considered to be equivocal; therefore fish embryo toxicity assay of these unique objects is avoided. However, the water of proglacial and moraine-dammed lakes could not be equated to standard laboratory media, as it is the unique admixture of inorganic and organic compounds determined by underlying bedrock and precipitation. The melt of permafrost, sub-moraine and rock-glacier ice contributes to an increase in the flux of major ions (Ca^2+^, Mg^2+^, SO_4_^2−^) and trace elements (Ni^2+^, Mn^2+^, Al^3+^, Hg^1+^, Pb^2+^) [47]. An expansion of zebrafish embryo assay to the monitoring of low-mineralized mountain waters may both significantly contribute to our understanding of embryonic adaptability and promote further ecotoxicological studies. The high-altitude lakes of the Northern Tien Shan provide unique opportunities for ecological research, as headwaters are heavily glaciated and in general accessible in a season. 

We undertook a series of bioassay research on 96 h exposure of *D. rerio* embryos to natural waters sampled from high-altitude lakes of two distinct mountain ranges: Ile Alatau and Dzungarian Alatau, NTSh. The toxicity status of lakes was assigned based on 10% and 50% response threshold in zebrafish embryo toxicity tests [1,48]. Of five Ile Alatau lakes three are proglacial (L. T-1, L. #1 and L. M. Mametova) and two are moraine-dammed: L. Titov fed by permafrost and snowcover melting and L. Akkol sourced by a high-mountain river. The chemical composition of studied lakes is comparable within (Table S1): sampled waters are low-mineralized, deficient in Ca, Mg and Cu ions, yet possess complicated ion composition (which is normal for mountainous areas with polymetallic ores), including Zn, Al, Se, and Mn and Mo concentrations in 3 to 5 times exceeding the control of maintenance water. The spatial variability of ion content is primarily related to the underlying lithology, while glacier, permafrost and snowcover melt contributions to runoff are second-order factors. Under the current analysis, in all Ile Alatau lakes Cd, Sr and Co, total petroleum hydrocarbons, lindane, and DDT were below detectable limits. The Pb was detected in samples from lakes Titov, M. Mametova, L. #1, and Akkol; however, below the MPC level for drinking water.

For the two proglacial lakes (L. M. Mametova and L. #1) and moraine-dammed L. Titov the incubation of zebrafish embryos for 96 h resulted in normal development and delayed hatching only (except for L. #1). Thus, *D. rerio* embryos are able to maintain normal development until hatching when incubated in low-mineralized media with diverse ionic composition, i.e., in ultra-fresh but natural water. The slight teratogenic effect has been observed under exposure to samples from L. Akkol (19.2%), the type of malformations detected were predominantly abnormalities of the spinal cord and growth retardations. Proglacial L. T-1 possesses slight embryotoxic (15.4%) and teratogenic (12.5%) effect, pericardial edema and tail malformations were most frequent malformations. The toxicity occurred at hatching. These two lakes stand out by pronounced deficiency in Cu, known to be essential for the metabolic reactions, development of fish bone and scale, but its relation to formation of abnormalities is controversial. It should be noted, that while Pb content in L. Akkol and L. #1 remained below MPC for drinking water, it reached and exceeded MPC for fishery water by 1.5-fold. At the same time, for L. Akkol slight teratogenicity was recorded while for L. #1 no effect was observed, suggesting, that either lead concentration was not effective and other contaminant is working or toxicity is altered by specific ionic interactions. Additional studies are needed to elucidate this mechanism.

In Dzungarian Alatau, the studied lakes are of very interesting disposition as they constitute the cascade of proglacial and moraine-dammed lakes beneath Tuyte Glacier, connected by superficial and underground channels. In addition, the studied area had no observed traces of any touristic or pastoral activity. The water samples proved to be quite distinct both by physicochemical analysis and bioassay tests. Compared to the Ile Alatau region, all lakes were characterized by lower mineralization. Supraglacial L. Tuyte-1 (which is actually the melted glacier) showed extremely low contents of Ca^2+^ and Mg^2+^ (undetectable), at the same time the level of Mn, Cu, Mo and Zn ions was high and even comparable to the control sample, the concentration of Pb was below MPC for drinking water, but reached MPC for fishery. The increased levels of TPHs were detected in all Tuyte lakes in accumulation pattern, so that MPC was 2 times elevated in L. Tuyte-1 and 8 times elevated in L. Tuyte-4. Generally the Dzungarian Alatau is under the influence of air masses driven from industrial and highly polluted North-East Kazakhstan regions; however, as detected TPHs pollution is a point estimate, it is preliminary to conclude that long-term atmospheric transport is a pollution source. The local emission from asphalt-and-concrete plant in lowland Taldykorgan city (Kazakhstan) or airflow from Xinxiang region (China) may be responsible for pollution as well.

The bioassay survey revealed the toxicity declining along the lake cascade: between the supraglacial L. Tuyte-1 and proglacial L. Tuyte-2 mortality and teratogenicity scores differ nearly twofold, intermediate L. Tuyte-3 proved to be slightly teratogenic and major moraine-dammed L. Tuyte-4 had no effect on survival and malformation rates in zebrafish embryos for 96 hpf. Coincidently, the water hardness and total mineralization increased gradually; however, so does the level of pollution by total petroleum hydrocarbons, which significantly complicates the case. 

Not taking into account the TPHs pollution and presence of lead, supraglacial lake Tuyte-1 represents the extreme medium for embryonic development as it is slightly acidic, highly deficient in Ca and lacks Mg ions. Growth retardations were frequent, and embryos were retarded at later stages; pericardial edemas were also frequent, severe and tended to expand to the yolk sac. The pericardial edema is often considered to be result of disruption in osmoregulation across the embryonic epidermis, but also if not too severe, it seems to be reversible [49]. Zebrafish embryos are able to maintain the osmotic balance through the functioning of specific chloride cells initially determined in the yolk-sac membrane, since 72 hpf the cutaneous ionocytes are responsible for active ion transport [50]. In case of L. Tuyte-1 embryos death was attributed to the hatching, survived larvae were weak and perished soon after hatching, we may presume that the chorion may mitigate the ionic composition of incubation media. On the other hand, it has been shown, that low hardness and pH may enhance the toxicity of heavy metals and some organic xenobiotics, including polycyclic aromatic hydrocarbons (PAHs), naphthoflavones, polychlorinated biphenyl 126 (PCB-126) [49,50,51]. The water of L. Tuyte-4 is also low-mineralized (10 mg/L) with significant Ca^2+^ and Mg^2+^ deficiency, so significant is the pollution with petroleum products; however, the survival and malformation rates in zebrafish embryos throughout 96 h of incubation were comparable to control group. Embryos were able to maintain normal development even in such extreme conditions, taking into account that levels of Cu^2+^ and Mn^2+^ are relatively high. 

The survey of *cyp1a* expression related to the xenobiotic biotransformation system may further clarify the case. The ability of zebrafish embryos to metabolize organic compounds via cytochrome P450 has been debated; however, it was shown that *CYP1A* transcript levels remain stable since 14 hpf, and prolongation of the exposure period to 96 h leads to embryo results similar to adult toxicity data [52]. The *cyp1a* gene is widely used as a biomarker of water pollution by the organic compounds including pharmaceuticals, pesticides, polycyclic aromatic hydrocarbons, organophosphates, dioxin and dioxin-like compounds, etc. [53,54,55]. In the present study a significant increase in *cyp1a* gene expression was demonstrated for Dzungarian Alatau lakes Tuyte-1 (10-fold), Tuyte-2 (7-fold) and Tuyte-3 (5-fold increase) (Table S2), the highest level of *cyp1a* gene expression directly correlated with malformation rate (*r* = 0.88) and negatively—with survival rate (*r* = −0.96). For the correlation analysis, we used the Pearson correlation function; although a four-parameter logistic function (4PL) is more suitable for such studies. However, due to the small number of data points (only four lakes in the cascade), constructing a sigmoid curve was impossible, so the correlation coefficients were determined using a linear function. Of course, this only allows us to obtain preliminary conclusions about the presence of correlations between the concentration of total petroleum hydrocarbons and the studied biological parameters.

Petroleum hydrocarbons are known to induce the expression of *cyp1a* gene [56] which may significantly contribute to toxicity through the toxification (i.e., products of biotransformation are more toxic than initial compound) and increased ROS production by metabolites and enzyme itself. At that inhibition of *cyp1a* expression may result in lower toxicity level [57]. In our study the *cyp1a* expression level negatively correlated with the increase in concentration of petroleum products (*r* = −0.998), thus either the threshold exists after which the pattern reverses or there is an agent that suppresses expression. In L. Tuyte-4 the *cyp1a* expression level dropped considerably, which is, we may propose, partially a reason for absence of toxic effect despite the high concentration of TPHs. In the Ile Alatau range the comparable decrease in *cyp1a* expression level has been observed in L. Titov only, lakes have in common the considerable share of snowpack and permafrost melt in the runoff, which is a significant source of recently precipitated pollutants. 

The expression level of two genes, *xpc* and *dpolb*, related to different types of excision repair was further investigated in hatched embryos, presuming that incubation in ultra-fresh natural water may result in the activation of only the first link of the DDR. It is known that *D. rerio* genome contains one copy of *xpc* gene, which is represented by three isoforms: transcripts 201, 202, and 203. The last transcript has no functional domains, since it consists of the first eight exons (out of 14), and its functions are currently unknown [31]. Two functional transcripts (201 and 202) differ in the absence of exon 7 in the transcript 202 protein. To assess the expression level of only the functional transcripts of the *xpc* gene, we designed primers for exons 8 and 9, which also excluded the possibility of genomic DNA contamination, since the intron size between these exons is 5142 nt. In zebrafish embryos *dpolb* is known to be involved in base-excision repair and double-strand break repair. *D. rerio* larvae have a generally reduced expression level of the *dpolb* gene compared to other reparation polymerases; however, starting from the 24 hpf stage, the expression level of this gene remains stable [58].

There was no obvious pattern in *xpc* and *dpolb* gene expression in hatched embryos (Table S2). For two lakes, non-toxic L. #1, and slightly toxic L. Akkol of Ile Alatau, the similar response was shown in significant increase in *xpc* expression concurrent with inhibition of *dpolb* expression. The induction of *xpc* gene alone was also detected for L. T-1, shown to possess slight toxic effect. The considerable enhancement of only *dpolb* gene expression was registered for L. Titov sample which is non-toxic to embryos. In Dzungarian Alatau lakes, induction of *xpc* has been registered in L. Tuyte-2 only, *dpolb* inhibition—in L. Tuyte-3. Incubation of zebrafish embryos in samples of L. Tuyte-4, which showed no detected developmental effects, resulted in a decrease in expression levels of all three genes, *xpc*, *dpolb* and *cyp1a*. We carried out the correlation analysis of the *dpolb* expression level dependence on the concentration of petroleum products in water samples and revealed the high inverse correlation (*r* = −0.92).

Data considering action of those genes in zebrafish embryos under xenobiotics exposure are scarce. The significant reduction in *dpolb* gene expression level in zebrafish embryos was evidenced by Lu et al. under the exposure to phthalate esters for 72 h. [58]. Phthalate esters are considered to be persistent environmental pollutants found worldwide in connection with petroleum pollution [59,60] and even may be synthesized by some freshwater algae [61]. There also are several studies on *D. rerio* where the graphene oxide has been shown to promote *dpolb* expression and to decrease the expression level of *cyp1a* [62,63]. So far, the nature of DNA-damaging agent(s) in studied water samples remains unclear, as the determination of specific xenobiotics requires the target analysis. As *cyp1a* level in Ile Alatau groups was comparable to control, a certain contaminant may work, which is toxic to DNA and does not undergo the CYP biotransformation. In addition, in the located in a close proximity Tuyuksu Glacier Miroshnikov et al., detected the radionuclides: an anthropogenic ^37^Cs, ^239+240^Pu, ^238^Pu, and ^241^Am and lithogenic ^235^U, ^238^U, and ^232^Th [15]. The alterations of DNA reparation system, detected by gene expression analysis can be estimated as markers of either intracellular disruptions or action of some non-common pollutant(s), or both, that are not immediately resulted in visible morphological effects. It should also be noted, that expression of the studied genes may be attributed to the later stages of development, 72–96 hpf.

In our experiments, in those groups where hatching at 72 hpf occurred in the majority of embryos (60–70%) the toxic effect was most pronounced. And in one group, L. M. Mametova which stands out with significantly delayed hatching, all other parameters including lethal and sublethal endpoints and genes expression level were comparable to the control. The chorion is known to play the considerable role in embryo protection from several xenobiotics working as effective or selective barrier and bind heavy metals [64,65]. Considering the outstanding role of chorion in maintenance of ionic balance in developing embryo, the hatched embryos may be more susceptible to the extreme water composition and/or pollutants action. For some high-altitude lakes we may also presume the presence of some undetermined compound(s), while in supraglacial lake low pH and ultra-low mineralization significantly contribute to overall water toxicity. The issue of antagonistic or synergistic action of individual pollutants under specific ionic water composition and their transformations is difficult to differentiate, but it should be considered. Low mineralization may significantly alter the processes of osmoregulation in zebrafish embryos making them more vulnerable to the co-existing chemical agents.

## 5. Conclusions

The bioassay of natural aquatic ecosystems is always a challenging area of research, at the same time helping to understand the limits of fish embryo adaptability. In the present work, we made an attempt to evaluate the toxic potential of several high-altitude lakes of Northern Tien Shan in order to assess the application of zebrafish embryo toxicity assay for low-mineralized water bodies. One of the main conclusions of the study is that *D. rerio* embryo toxicity test is quite feasible from a methodological perspective for the ecotoxicological monitoring of ultra-fresh natural waters. Despite the fact, that the studied water samples had mineralization levels ranging from 10 to 70 mg/L, while the recommended values are equivalent to 100–300 mg/L CaCO_3_, the analysis of lethal and sublethal endpoints revealed that zebrafish embryos are able to maintain normal development, at least until hatching. For proglacial and moraine-dammed lakes the water composition is the whole spectrum of interaction between rock-earth substrate and melting cryosphere; the deficiency in ions fundamental for the embryogenesis is probably mitigated by the complicated ion composition of water samples. The issue of application of zebrafish embryo assay to the monitoring of extreme medium such as supraglacial lakes is questionable. The expression levels of the *xpc*, *dpolb*, and *cyp1a* genes varied across all studied high-altitude lakes; however, we were able to show that the evaluation of the gene expression levels of *xpc* and *dpolb* related to the DNA reparation system and *cyp1a* related to biotransformation processes is reliable and sensitive biomarker of metabolic responses beyond sublethal endpoints and is helpful for understanding the specificity of embryos response to toxic agent(s). The issue of ecotoxicological monitoring of glaciated areas should not be entirely concentrated on heavy metals and common organic pollutants, but should also cover radionuclides, phthalates, graphene oxide, micro-and nanoplastics, and others, as much as possible. 

The results of the current bioassay of high-altitude lakes raise several important issues that deserve further attention: (a) application of prolonged fish embryo toxicity test, i.e., up to 144 hpf and genotoxicity assessment with further studies on genes related to the DNA reparation system; (b) parallel experiments with pure and adjusted (by divalent ions and pH) natural waters of supraglacial and proglacial lakes to distinguish xenobiotics-related toxicity; (c) application of zebrafish embryo toxicity assay to high-altitude lake sediments.

## Figures and Tables

**Figure 1:**
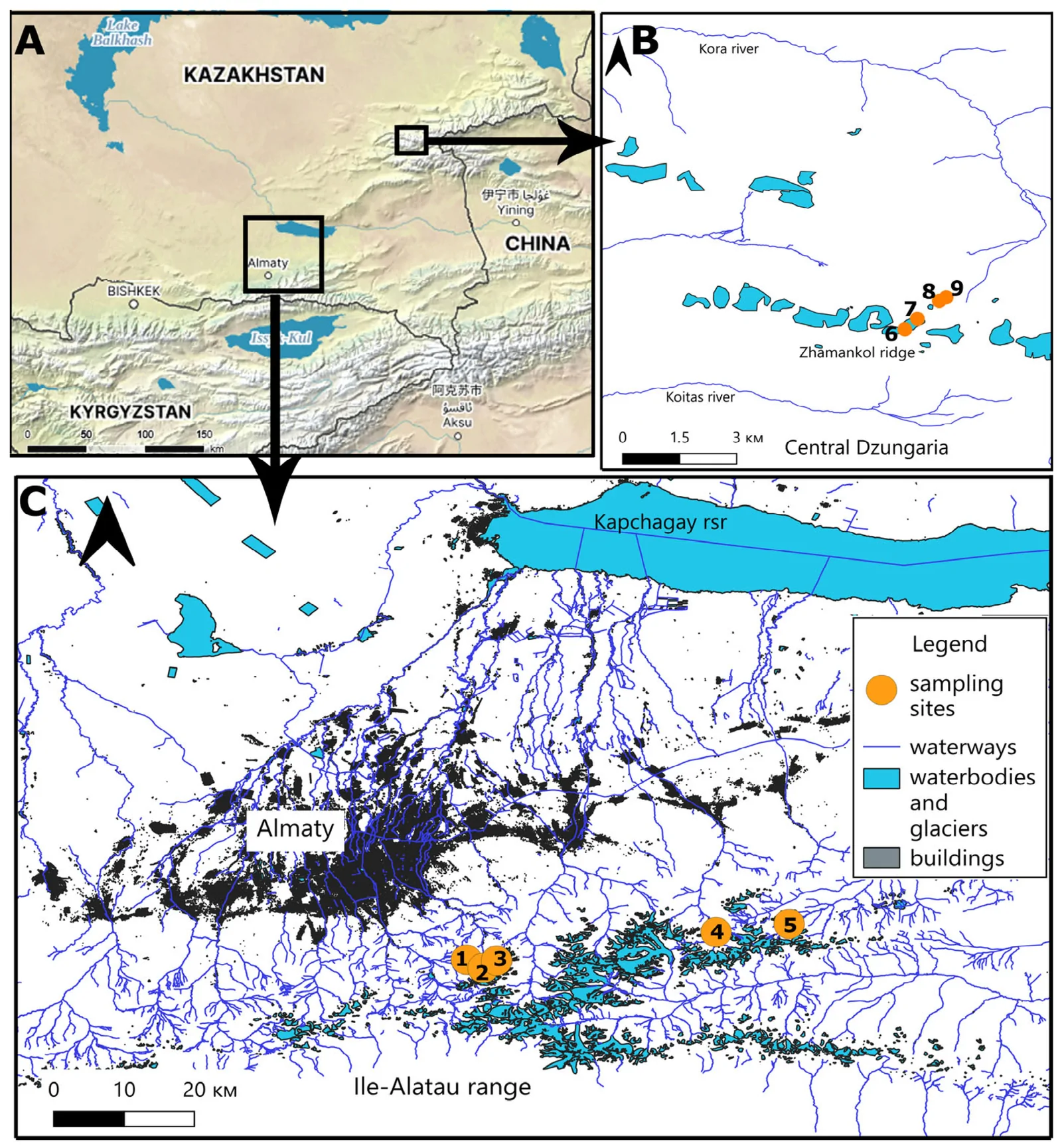
Location of the studied high-altitude lakes (sampling sites) in the Northern Tien Shan, Kazakhstan. (**A**) general location of research areas; (**B**) lakes (L.) in Dzungarian Alatau: 6—L. Tuyte-1, 7—L. Tuyte-2, 8—L. Tuyte-3, 9—L. Tuyte-4; (**C**) lakes (L.) in Ile Alatau, 3111-3560 m a.s.l.: 1—L. Titov, 2—L. T-1, 3—L. M. Mametova, 4—L. Akkol, 5—L. #1.

**Figure 2:**
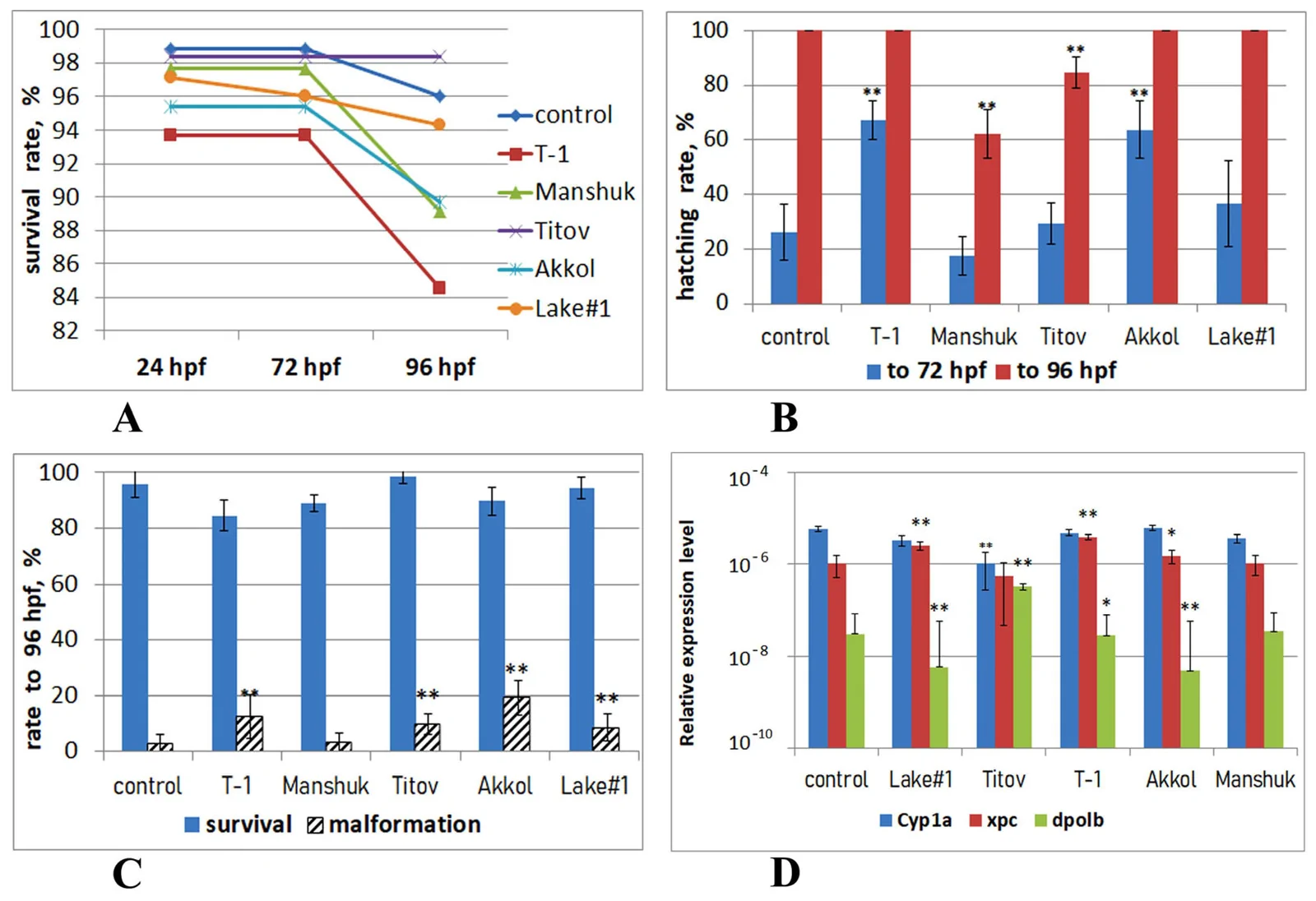
The overview of lethal and sub-lethal endpoints in *D. rerio* embryos exposed to natural water samples from high-altitude lakes (3111–3560 m a.s.l.) of the Ile Alatau range, NTSh, for 96 h. (**A**) Dynamics of embryo survival up to 96 hpf; (**B**) hatching by 72 and 96 hpf; (**C**) survival and developmental abnormalities at 96 hpf; all rates for each group are given as the mean for five independent runs each with 25 fertilized embryos initially, ±SD; (**D**) Relative gene expression levels in 96 h larvae: relative mRNA expression of cytochrome P 450 1a (*cyp1a*), Xeroderma Pigmentosum complementation group C (*xpc*), DNA polymerase β (*dpolb*). The bars represent the mean standard deviation. One asterisk (*) indicates statistically significant difference (α ≤ 0.05) between control and treated group using Mann–Whitney U test for independent samples. Two asterisks (**) indicate statistically significant differences (α ≤ 0.05) between control and treated group with high statistical test power (1-β ≥ 0.8).

**Figure 3:**
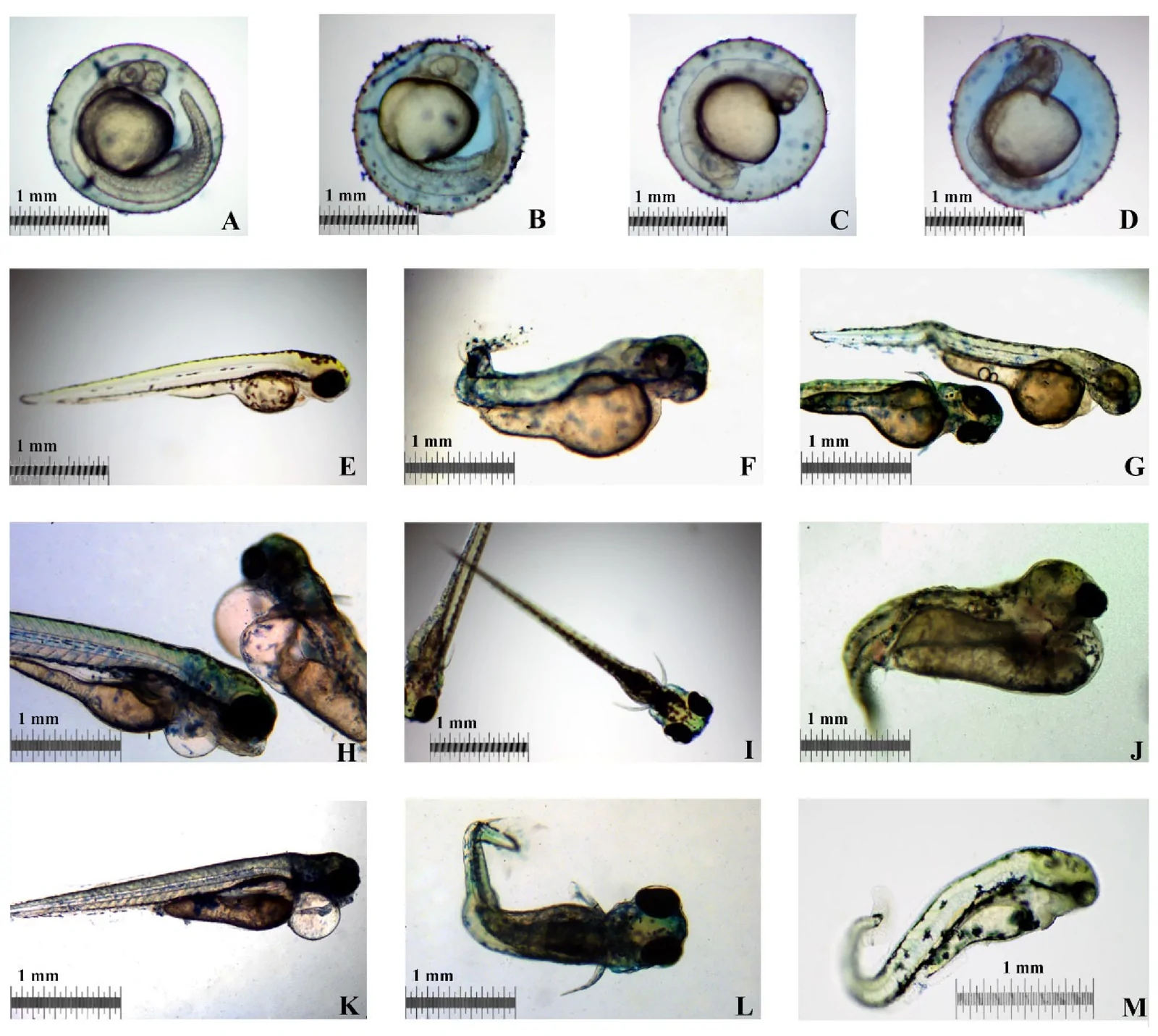
Representative images of *D. rerio* embryos exposed to natural surface water from high-altitude lakes of NTSh. (**A**) control, 24 hpf; (**B**) L. Titov, normal development, 24 hpf; (**C**) L. Tuyte-1, severe malformations, 24 hpf; (**D**) L. Tuyte-2, severe malformations, 24 hpf; (**E**) L. #1, normal development, 72 hpf; (**F**) L. T-1, tail malformation, 72 hpf; (**G**) L. M. Mametova, tail malformation, 72 hpf; (**H**) L. T-1, pericardial and yolk edemas, 72 hpf; (**I**) L. Tuyte-4, normal development, 96 hpf; (**J**) L. Akkol, severe axial malformations, 96 hpf; (**K**) L. Tuyte-3, pericardial edema, 96 hpf; (**L**) L. Tuyte-2, underdeveloped and malformed tail, 96 hpf; (**M**) L. Tuyte-1, growth retardation and tail malformation, 96 hpf.

**Figure 4:**
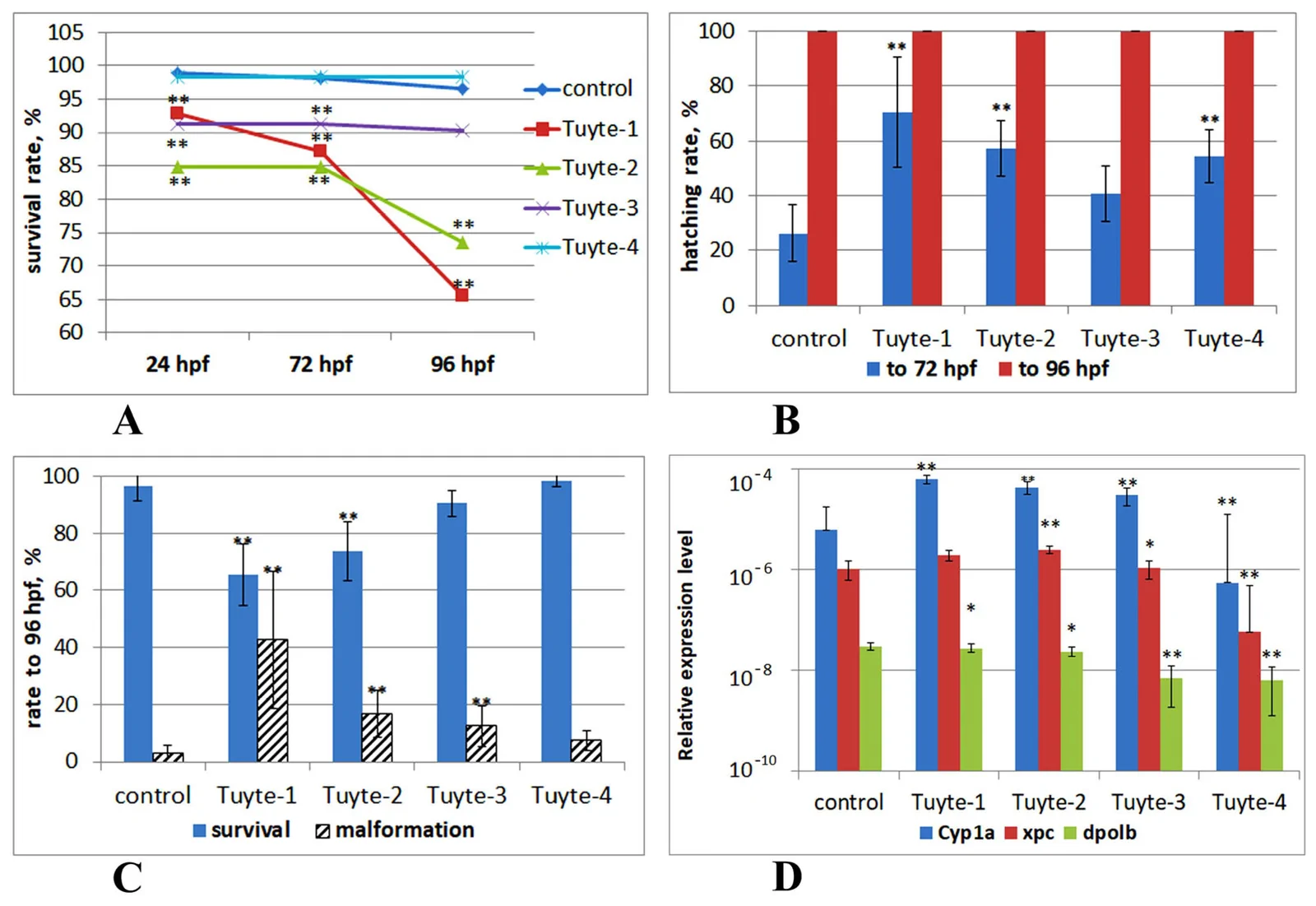
The overview of lethal and sub-lethal endpoints in *D. rerio* embryos exposed to natural water samples from high-altitude lakes (3070–3325 m a.s.l.) of Dzungarian Alatau, NTSh, for 96 h. (**A**) Dynamics of embryo survival for 96 hpf; (**B**) hatching by 72 and 96 hpf; (**C**) survival and developmental abnormalities at 96 hpf; all rates for each group are given as the mean for five independent runs each with 25 fertilized embryos initially, ±SD; (**D**) Relative gene expression levels in 96 h larvae: relative mRNA expression of cytochrome P 450 1a (*cyp1a*), Xeroderma Pigmentosum complementation group C (*xpc*), DNA polymerase β (*dpolb*). The bars represent the mean standard deviation. One asterisk (*) indicates statistically significant difference (α ≤ 0.05) between control and treated group using Mann–Whitney U test for independent samples. Two asterisks (**) indicate statistically significant differences (α ≤ 0.05) between control and treated group with high statistical test power (1 − β ≥ 0.8).

**Table 1:** Brief characteristics of the studied high-altitude lakes of NTSh.

Formal Name/ (Author’s Designation) *	Coordinates	Catchment Area	Elevation, m a.s.l.	Lake Type **
Moraine lake no.1/(L. #1)	43°8′41.51″ 77°36′46.13″	Turgen River Ile Alatau	3560	proglacial (Gorniy Institute Glacier)
Lake Akkol/(L. Akkol)	43°7′47.61″ 77°29′4.61″	Issyk River Ile Alatau	3111	moraine-dammed
Lake Titov/(L. Titov)	43°4′48.42″ 77°2′48.84″	Kishi Almaty River Ile Alatau	3320.	moraine-dammed
Lake of T-1 glaciological station/(L. T-1)	43°3′53.87″ 77°4′34.41″	Kishi Almaty River Ile Alatau	3500	proglacial (Molodezhniy Glacier)
Lake Manshuk Mametova/(L. M. Mametova)	43°4′42.05″ 77°6′0.05″	Kishi Almaty River Ile Alatau	3550	proglacial (M. Mametova Glacier)
No name/(L. Tuyte-1)	44°55′2.07″ 79°11′53.67″	Kora River Dzungarian Alatau	3321	supraglacial (Tuyte Glacier)
No name/(L. Tuyte-2)	44°55′15.14″ 79°12′8.65″	Kora River Dzungarian Alatau	3110	proglacial (Tuyte glacier)
No name/(L. Tuyte-3)	44°55′37.17″ 79°12′34.99″	Kora River Dzungarian Alatau	3068	moraine-dammed
No name/(L. Tuyte-4)	44°55′41.60″ 79°12′43.36″	Kora River Dzungarian Alatau	3020	moraine-dammed

* the name of the lake as it is indicated in the article is given in brackets. ** definitions of all the specified types of lakes are given in Section “Abbreviations”.

## Data Availability

The original contributions presented in the study are included in the article. All the data included in this article are publicly available. Further inquiries can be directed to the corresponding author.

## Supplementary Materials

The following supporting information can be downloaded at: https://www.mdpi.com/article/10.3390/biology15191718/s1, Figure S1: Precise location and images of studied high-mountain lakes of Ile Alatau, Northern Tien Shan, Kazakhstan; Figure S2: Precise location and images of studied high-mountain lakes of Dzungarian Alatau, Northern Tien Shan, Kazakhstan; Table S1: Results of physicochemical analysis of surface water samples from high-mountain lakes of NTSh; Figure S3: qPCR results for two total RNA samples of *D. rerio* and the corresponding melting curves; Figure S4: Efficiency plots of qPCR on a series of cDNA dilutions generated in Q-Gene96 program for genes: (A) *cyp1a*; (B) *xpc*; (C) *dpolb*; (D) 18S rRNA; Table S2: Summary on relative expression level of target genes *cyp1a*, *xpc* and *dpolb* in *D. rerio* embryos after 96 h exposure to natural surface water of high-mountain lakes of Northern Tien Shan.

## Abbreviations

The following terms and abbreviations are used in this manuscript:

GOST	Technical standards maintained by EASC
hpf	Hours post fertilization
NTSh	Northern Tien Shan
MPC	Maximum Permissible Concentration
TPHs	Total Petroleum Hydrocarbons
high-altitude lake	A lake positioned at or above the regional tree line, typically exceeding 3000 m a.s.l.
moraine-dammed lake	A lake which water body is impounded by an accumulation of unconsolidated glacial debris deposited at a past or present glacier margin.
proglacial lake	A body of water formed immediately adjacent to, or in direct contact with, the terminus of a glacier
supraglacial lake	A dynamic, ephemeral body of meltwater that forms directly on top of a glacier’s surface.
