# Supplementary material for: Assessing Low-Mineralized Surface Water via Danio rerio Embryo Toxicity Test: A Case Study of Glacial Lakes in the Northern Tien Shan
Source: Biology (Basel). 2026 Sep 30;15(19):1718. doi: 10.3390/biology15191718 (PMC13663472; doi:10.3390/biology15191718)
Supplement: Supplementary file 1 [file biology-15-01718-s001.zip › biology-4509965-supplementary.pdf]

**Supplements**

# Assessing Low-Mineralized Surface Water via *Danio rerio* Embryo Toxicity Test: A Case Study of Glacial Lakes in the Northern Tien Shan

**Mariya A. Suvorova [1,*], Anna S. Nizkorodova [2] and Alina A. Dmitriyeva [1]**

[1] Department of Genetics and Molecular Biology, Faculty of Biology and Biotechnology, Al-Faraby Kazakh National University, Almaty 050040, Kazakhstan; dmitriyeva.alin@gmail.com
[2] M. Aitkhozhin Institute of Molecular Biology and Biochemistry, Almaty 050012, Kazakhstan; anna_niz@yahoo.com
* Correspondence: suvorova.maryiya@kaznu.kz or masyanskii@gmail.com; Tel.: +7-(705)-101-9080

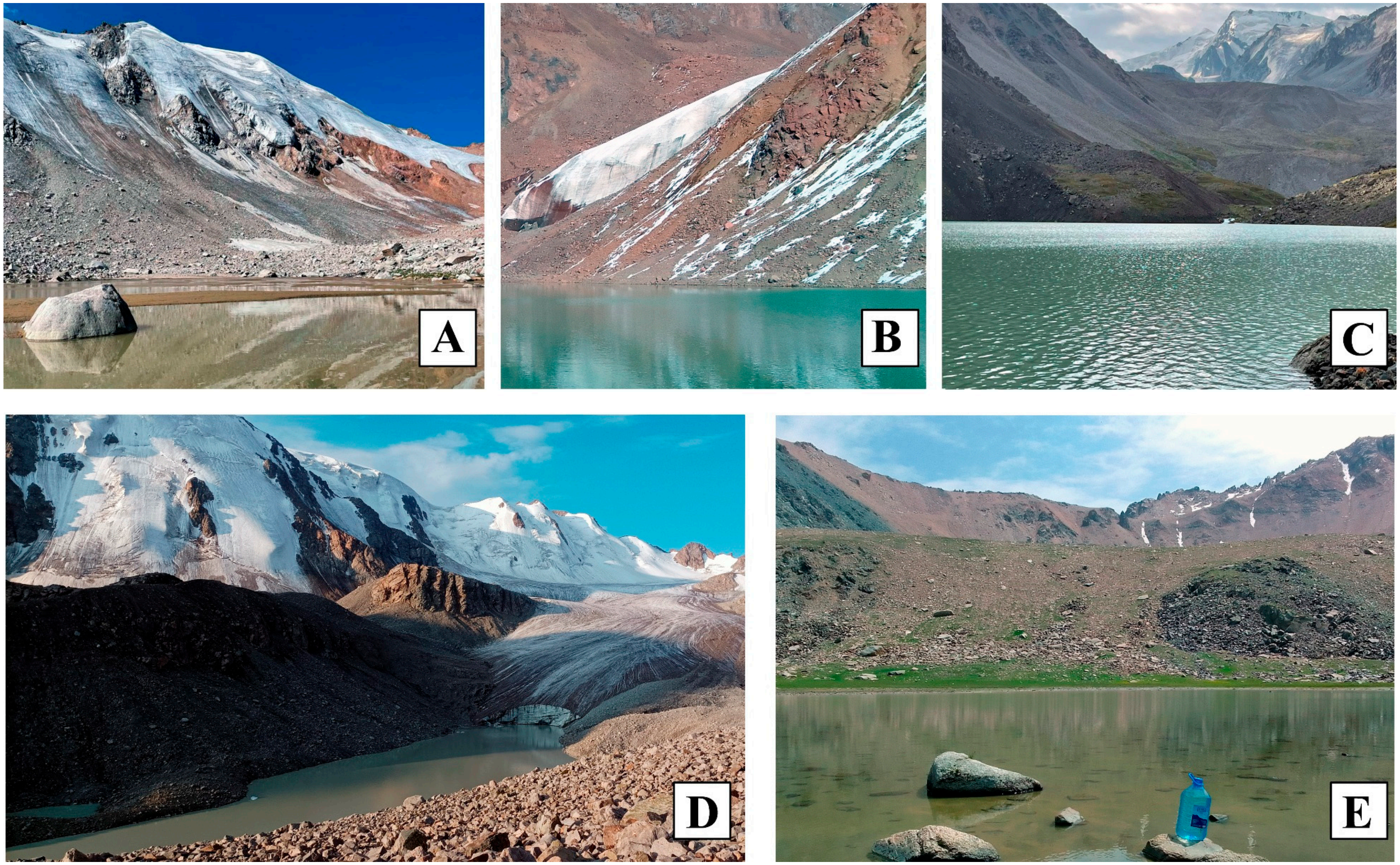


**Figure S1.** Precise location and images of studied high-mountain lakes of Ile Alatau, Northern Tien Shan, Kazakhstan: A) L.T-1 (3500 m a.s.l.) 43°3'53.87" 77°4'34.41"; B) L.M.Mametova (3550 m a.s.l.) 43°4'42.05" 77°6'0.05"; C) L.Akkol (3111 m a.s.l.) 43°7'47.61" 77°29'4.61"; D) L#1 (3560 m a.s.l.) 43°8'41.51" 77°36'46.13"; E) L.Titova (3320 m a.s.l.) 43°4'48.42" 77°2'48.84"

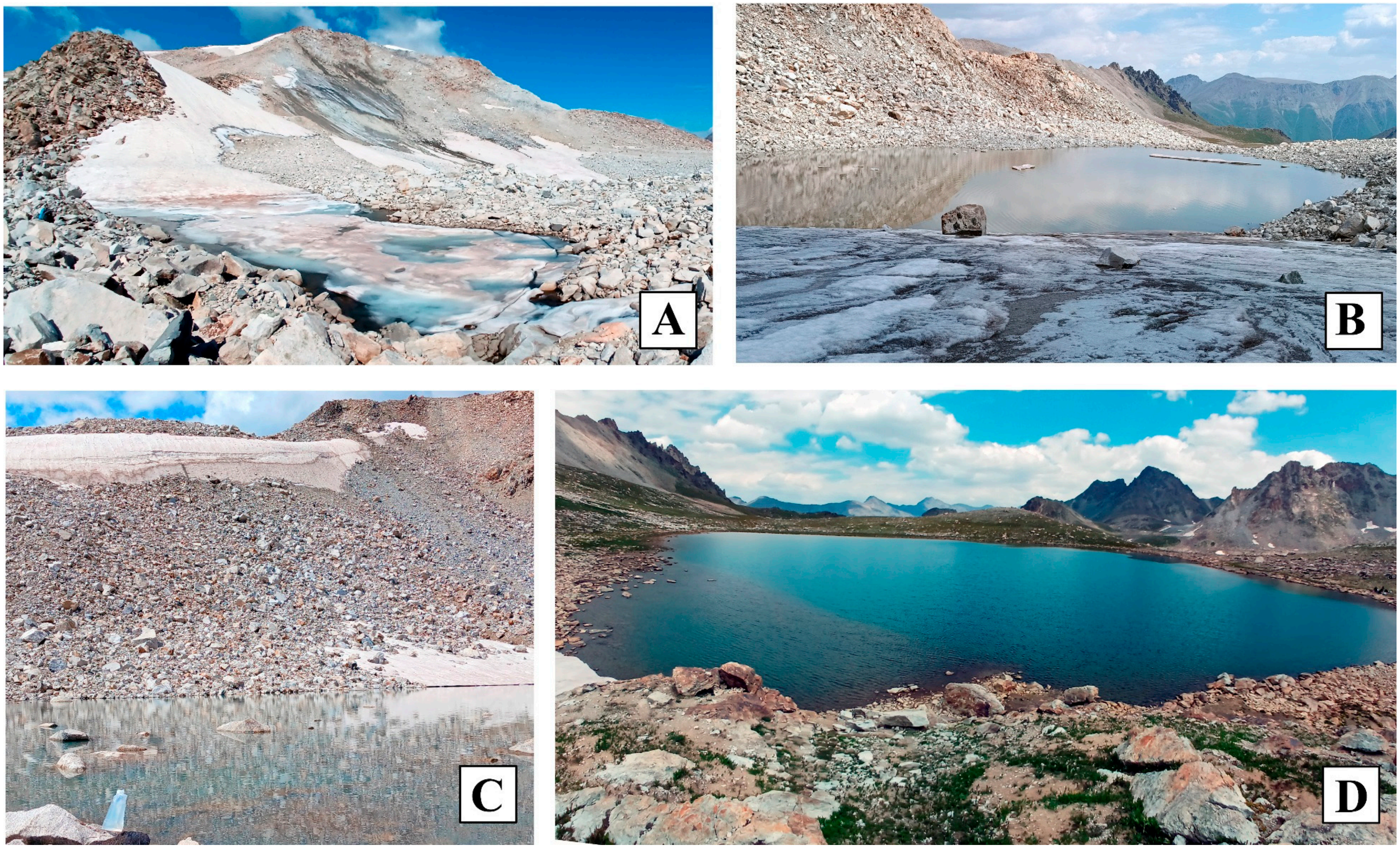


**Figure S2.** Precise location and images of studied high-mountain lakes of Dzungarian Alatau, Northern Tien Shan, Kazakhstan: A) L.Tuyte-1 (3321 m a.s.l.) 44°55'2.07" 79°11'53.67"; B) L.Tuyte-2 (3110 m a.s.l.) 44°55'15.14" 79°12'8.65"; C) L.Tuyte-3 (3068 m a.s.l.) 44°55'37.17" 79°12'34.99"; D) L.Tuyte-4 (3020 m a.s.l.) 44°55'41.60" 79°12'43.36"

**Table S1.** Results of physicochemical analysis of surface water samples from high-mountain lakes of NTSh

| Parameter | Control (maintenance water) | Ile Alatau lakes | | | | | Dzungarian Alatau lakes | | | | MPC |
|---|---|---|---|---|---|---|---|---|---|---|---|
| | | Akkol | Lake#1 | T-1 | M.Mametova | Titov | Tuyte-1 | Tuyte-2 | Tuyte-3 | Tuyte-4 | |
| Mineralization, mg/L | 108 | 70 | 61 | 46 | 65 | 43 | 10 | 10 | 10 | 10 | <1000 |
| Dry residue, mg/L | 74 | 46 | 39 | 30 | 41 | 27 | 9 | 8 | 7 | 7 | <1500 |
| Total hardness, mM/L | 1,36 | 0,85 | 0,7 | 0,6 | 0,85 | 0,55 | 0,05 | 0,05 | 0,05 | 0,1 | <7.0 |
| Hydrocarbonate, mg/L | 51,9 | 39,7 | 36,6 | 27,5 | 39,7 | 27,5 | 3,1 | 3,9 | 5,0 | 6,1 | - |
| Chlorides, mg/L | 13,1 | 1,0 | 2,1 | 1,0 | 1,1 | 1,0 | 1,8 | 1,0 | 0,4 | 0,4 | <350 |
| Sulfates, mg/L | 8,2 | 8,0 | 4,1 | 3,1 | 6,0 | 2,1 | 1,0 | 1,0 | 1,0 | 1,0 | <500 |
| Nitrates, mg/L | 1,0 | 1,0 | 2,0 | 2,0 | 2,0 | 1,0 | - | - | - | - | <45 |
| Fluorides, mg/L | 0,6 | 0,5 | 0,3 | 0,3 | 0,1 | 0,4 | 0,3 | - | - | - | <1.5 |
| pH | 7,7 | 7,0 | 6,99 | 6,92 | 6,6 | 6,93 | 6,04 | 6,3 | 6,48 | 6,48 | 6-9 |
| Sodium, mg/L | 4,2 | 2,0 | 1,5 | 0,7 | 1,2 | 0,7 | 0,8 | 0,6 | 0,6 | 0,6 | <200 |
| Potassium, mg/L | 1,2 | 0,9 | 1,4 | 0,5 | 0,6 | 0,5 | 1,8 | 1,1 | 0,1 | 0,1 | - |
| Cadmium, mg/L | ND | ND | ND | ND | ND | ND | ND | ND | ND | ND | <0.001 |
| Calcium, mg/L | 20,2 | 14,0 | 10,0 | 8,0 | 9,0 | 7,0 | 1,0 | 1,0 | 1,0 | 1,0 | - |
| Magnesium, mg/L | 4,3 | 1,8 | 2,4 | 2,4 | 4,9 | 2,4 | ND | 0,6 | 0,6 | 0,6 | - |
| Aluminum, mg/L | ND | ND | 0,080 | 0,030 | 0,060 | 0,008 | ND | ND | ND | ND | <0.5 |
| Cobalt, mg/L | ND | 0,0013 | 0,0008 | ND | ND | ND | ND | ND | ND | ND | <0.1 |
| Manganese, mg/L | 0,0059 | 0,0083 | 0,017 | 0,0057 | 0,0088 | 0,0039 | 0,0053 | 0,0048 | 0,0048 | 0,0048 | <0.1 |
| Copper, mg/L | 0,01 | 0,0018 | 0,0078 | 0,0023 | 0,0061 | 0,0049 | 0,01 | 0,007 | 0,0039 | 0,0039 | <1.0 |
| Molybdenum, mg/L | 0,0012 | 0,01 | 0,0061 | 0,0034 | 0,0067 | 0,004 | 0,0012 | - | - | - | <0.25 |
| Nickel, mg/L | 0,01 | 0,0012 | 0,0022 | 0,0032 | 0,0043 | 0,0019 | 0,01 | 0,009 | 0,008 | 0,0079 | <0.1 |
| Lead, mg/L | 0,0018 | 0,0093 | 0,0081 | - | 0,0030 | 0,0008 | 0,0089 | ND | ND | ND | <0.03 |
| Selenium, mg/L | ND | 0,0019 | 0,0062 | 0,005 | 0,0015 | 0,0081 | ND | ND | ND | ND | <0.01 |
| Silver, mg/L | ND | ND | ND | ND | ND | ND | 0,0007 | ND | ND | ND | <0.05 |
| Chromium, mg/L | 0,0054 | 0,0010 | 0,0001 | ND | ND | ND | 0,0075 | ND | ND | ND | <0.5 |
| Zinc, mg/L | 0,0037 | 0,0039 | 0,0058 | 0,0034 | 0,0062 | 0,0060 | 0,0031 | 0,0013 | 0,0013 | 0,0013 | <5.0 |
| Total petroleum hydrocarbons, mg/L | 0,011 | ND | ND | ND | ND | ND | 0,266 | 0,41 | 0,585 | 0,799 | <0.1 |
| Phenolic index, mg/L | ND | ND | ND | ND | ND | ND | 0,0003 | 0,0005 | 0,0008 | 0,0008 | <0.25 |
| NB. MPC – Maximum Permissible Concentration, for drinking water | | | | | | | | | | | |

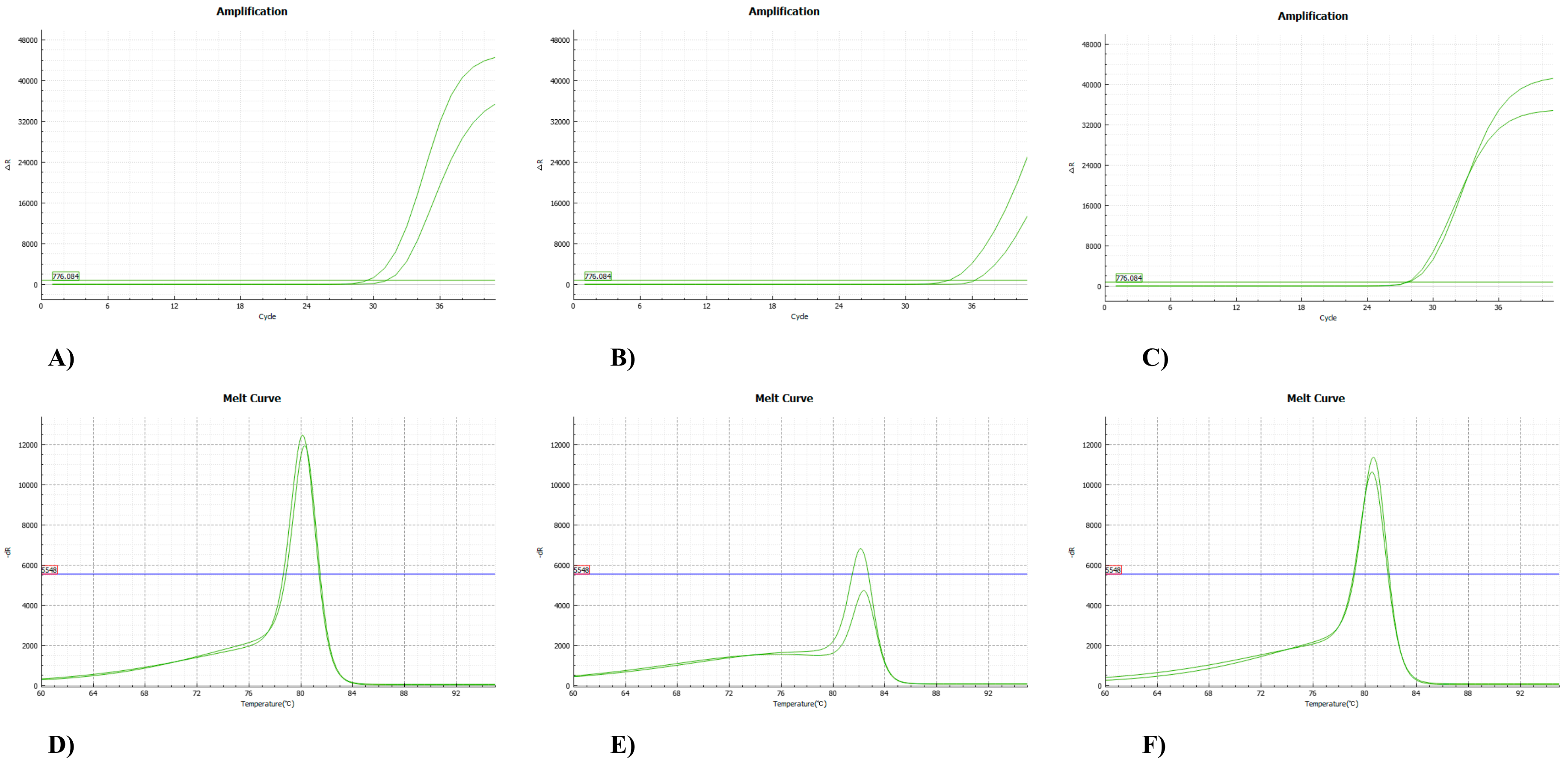


**Figure S3.** qPCR results for two total RNA samples of *D. rerio* and the corresponding melting curves: A) and D) Ct values and melting curves for *dpolb*; B) and E) Ct values and melting curves for *cyp1a*; C) and F) Ct values and melting curves for *xpc*.

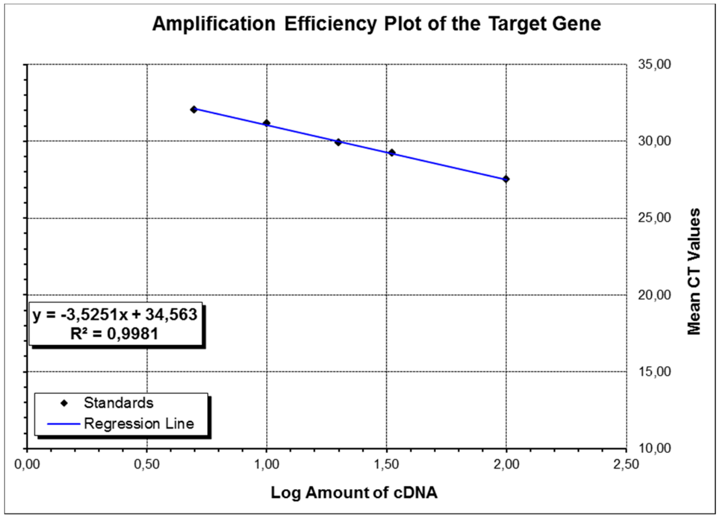


**A)**

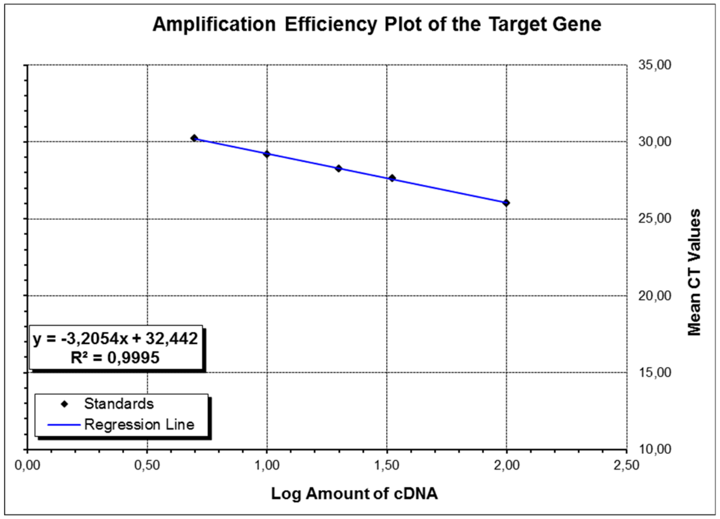


**B)**

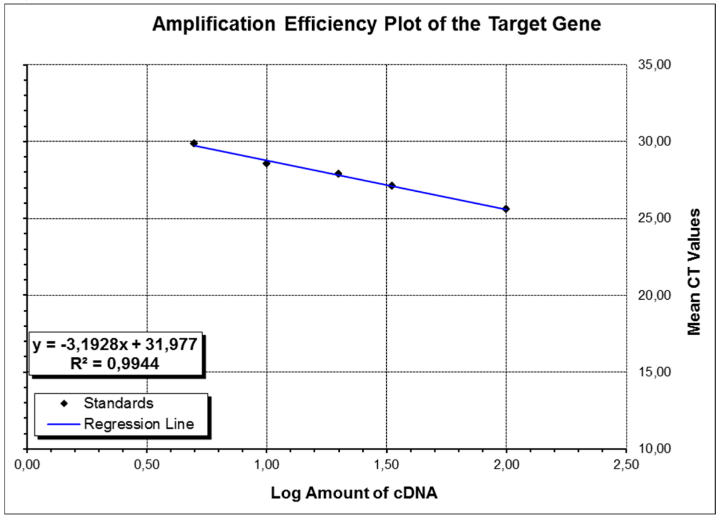


**C)**

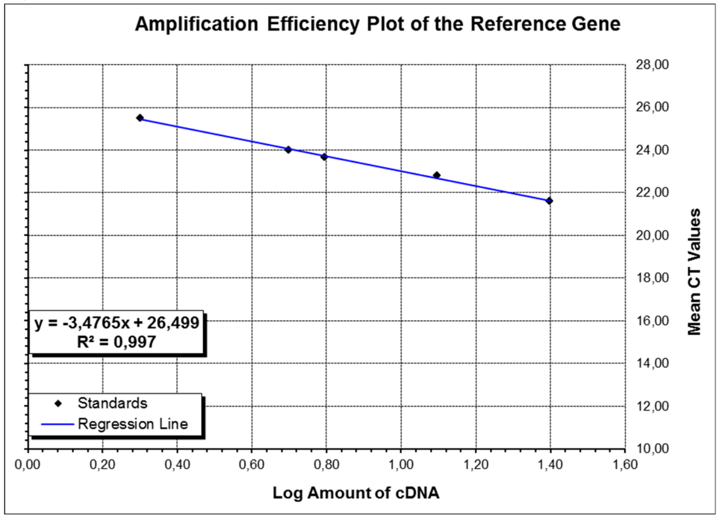


**D)**

**Figure S4.** Efficiency plots of qPCR on a series of cDNA dilutions generated in Q-Gene96 program for genes: A) *cyp1a*; B) *xpc*; C) *dpolb*; D) 18S rRNA. Number of repeats for each gene $n = 6$.

**Table S2**. Summary on relative expression level of target genes *cyp1a*, *xpc* and *dpolb* in *D.rerio* embryos after 96 h exposure to natural surface water of high-mountain lakes of Northern Tien Shan

| sample | Expression level | | | | | | | | |
|---|---|---|---|---|---|---|---|---|---|
| | *cyp1a* | | | *xpc* | | | *dpolb* | | |
| | to control | Statistics | | to control | Statistics | | to control | Statistics | |
| | | p | 1-β | | p | 1-β | | p | 1-β |
| **Heavy metals** | | | | | | | | | |
| Pb 3 mg/L | 250% | 3.2E-04 | 0.9 | 652% | 8E-06 | 0.9 | 161% | 0.01 | 0.6 |
| Cd 50 µg/L | 167% | 0.04 | 0.8 | 208% | 3.1E-04 | 0.8 | 66% | 0.01 | 0.5 |
| **Ile Alatau** | | | | | | | | | |
| Lake#1 | 55% | 0.37 | 0.7 | 246% | 1.4E-03 | 0.8 | 19% | 0.05 | 0.8 |
| Akkol | 105% | 0.37 | 0.6 | 147% | 8.4E-05 | 0.6 | 16% | 0.05 | 0.8 |
| Titov | 18% | 0.04 | 0.8 | 54% | 0.21 | 0.7 | 1100% | 0.04 | 0.8 |
| T-1 | 82% | 0.15 | 0.6 | 383% | 0.04 | 0.8 | 92% | 0.05 | 0.6 |
| M.Mametova | 61% | 0.26 | 0.6 | 104% | 0.17 | 0.5 | 113% | 0.07 | 0.5 |
| **Dzungarian Alatau** | | | | | | | | | |
| Tuyte-1 | 1044% | 4E-05 | 0.9 | 185% | 0.07 | 0.6 | 92% | 0.01 | 0.5 |
| Tuyte-2 | 713% | 5.4E-05 | 0.9 | 242% | 3.4E-03 | 0.9 | 79% | 0.01 | 0.5 |
| Tuyte-3 | 494% | 3.97E-05 | 1.0 | 103% | 0.04 | 0.5 | 23% | 0.03 | 0.8 |
| Tuyte-4 | 9% | 0.05 | 0.9 | 6% | 0.04 | 0.8 | 21% | 0.05 | 0.8 |
| The expression level is shown as a percentage relative to the expression of the control group. Shaded cells represent values that are statistically significant ($p \leq 0.05$) and have high test power ($1-\beta \geq 0.8$). | | | | | | | | | |
